# Early *Neospora caninum* infection dynamics in cattle after inoculation at mid-gestation with high (Nc-Spain7)- or low (Nc-Spain1H)-virulence isolates

**DOI:** 10.1186/s13567-019-0691-6

**Published:** 2019-09-24

**Authors:** Laura Jiménez-Pelayo, Marta García-Sánchez, Patricia Vázquez, Javier Regidor-Cerrillo, Pilar Horcajo, Esther Collantes-Fernández, Javier Blanco-Murcia, Daniel Gutiérrez-Expósito, Alicia Román-Trufero, Koldo Osoro, Julio Benavides, Luis Miguel Ortega-Mora

**Affiliations:** 1 Saluvet, Animal Health Department, Faculty of Veterinary Sciences, Complutense University of Madrid, Ciudad Universitaria s/n, 28040 Madrid, Spain; 2 Saluvet-innova, Faculty of Veterinary Sciences, Complutense University of Madrid, Ciudad Universitaria s/n, 28040 Madrid, Spain; 3 Department of Animal Medicine and Surgery, Faculty of Veterinary Sciences, Complutense University of Madrid, Ciudad Universitaria s/n, 28040 Madrid, Spain; 4 Instituto de Ganadería de Montaña (CSIC-Universidad de León), 24346 León, Spain; 5 Regional Service for Research and Agri-Food Development (SERIDA), 33300 Villaviciosa, Asturias, Spain

## Abstract

Early *Neospora caninum* infection dynamics were investigated in pregnant heifers intravenously inoculated with PBS (G-Control) or 10^7^ tachyzoites of high (G-NcSpain7)- or low (G-NcSpain1H)-virulence isolates at 110 days of gestation. Serial culling at 10 and 20 days post-infection (dpi) was performed. Fever was detected at 1 dpi in both infected groups (*P* < 0.0001), and a second peak was detected at 3 dpi only in G-NcSpain7 (*P* < 0.0001). At 10 dpi, Nc-Spain7 was detected in placental samples from one animal related to focal necrosis, and Nc-Spain7 transmission was observed, although no foetal lesions were associated with this finding. The presence of Nc-Spain1H in the placenta or foetuses, as well as lesions, were not detected at 10 dpi. At 20 dpi, G-NcSpain7 animals showed almost 100% positive placental tissues and severe focal necrosis as well as 100% transmission. Remarkably, foetal mortality was detected in two G-NcSpain7 heifers. Only one animal from G-NcSpain1H presented positive placental samples. No foetal mortality was detected, and lesions and parasite transmission to the foetus were not observed in this group. Finally, 100% of G-NcSpain7 heifers at 20 dpi presented specific antibodies, while only 60% of G-NcSpain1H animals presented specific antibodies at 20 dpi. In addition, earlier seroconversion in G-Nc-Spain7 was observed. In conclusion, tachyzoites from Nc-Spain7 reached the placenta earlier and multiplied, leading to lesion development, transmission to the foetus and foetal mortality, whereas Nc-Spain1H showed delayed infection of the placenta and no lesional development or transmission during early infection.

## Introduction

*Neospora caninum* is an apicomplexan protozoan parasite that is considered to be one of the main causes of abortion in cattle. Horizontal transmission via oocyst ingestion is possible, although transplacental transmission in cattle seems to be the most efficient infection route [1]. In pregnant cattle, infection with this parasite may lead to abortion, birth of still-born calves, birth of new-born calves with clinical signs or birth of clinically healthy but persistently infected calves [2, 3].

The disease outcome is influenced by several factors, including the maternal immune response in the placenta and the relative immunocompetence of the foetus at the time of infection, which are two key variables [4, 5]. Experimental *N. caninum* infection in pregnant cattle during the first term generally produces foetal death and abortion, and foetuses show more severe lesions [6, 7]. Experimental infection from the second trimester onward, which is when the foetal immune system begins to develop, generally results in clinically healthy but congenitally infected calves [6, 8–10], although infection with the highly virulent isolate Nc-Spain7 induced at least 50% foetal death at 110 days of gestation (dg) [11, 12]. Under natural conditions, abortion caused by *N. caninum* is more frequent during the second trimester of pregnancy [13, 14].

A limited number of studies have been conducted to investigate the consequences of *N. caninum* infection at mid-gestation [9, 11, 12, 15, 16]. Recently, intravenous (IV) inoculation of 10^7^ tachyzoites of Nc-Spain7 at mid-gestation produced 50% foetal death until 42 days post-infection (dpi) [11] and 66.6% foetal death when gestation lasted until term. Moreover, foetal death was observed using lower doses of Nc-Spain7 tachyzoites, although a lower percentage of abortions and a delayed presentation were detected as the dose decreased [12].

The outcome of the infection in pregnant cattle also depends on the isolate. Specifically, the high-virulence isolate Nc-Spain7 showed a percentage of abortion and vertical transmission of 100% in a bovine model at early gestation [7, 17], whereas the infection in experimentally infected cattle with the low-virulence isolate Nc-Spain1H did not result in foetal death [18].

In the current study, the aim was to investigate how the differences between high (Nc-Spain7)- and low (Nc-Spain1H)-virulence isolates of *N. caninum* influence the clinical outcome, parasite distribution and burden, lesion development in placental and foetal tissues, and the specific antibody response during early infection in pregnant heifers inoculated at mid-gestation. The lack of bovine models studying early infection and the lack of experimental infections comparing isolates make the implementation of this model necessary to elucidate the pathogenesis of bovine neosporosis at mid-gestation, which is when most abortions occur in naturally infected cattle [19, 20].

## Materials and methods

### Animals and experimental design

Asturiana heifers, aged 20–30 months, were selected after assessing their seronegativity to *N. caninum*, Infectious Bovine Rhinotracheitis (IBR) virus, Bovine Viral Diarrhoea (BVD) virus, *Leptospira* and *Mycobacterium avium* subsp. *paratuberculosis* by ELISA. The health and reproductive management of the animals is detailed in Additional file 1. Pregnant heifers (*n* = 24) were randomly distributed in three experimental groups, G-Control (*n* = 6), G-NcSpain7 (*n* = 9) and G-NcSpain1H (*n* = 9) and inoculated intravenously at 110 days of gestation with phosphate buffered saline (PBS) and 10^7^ culture-derived tachyzoites of Nc-Spain7 and Nc-Spain1H isolates, respectively. Three animals from G-Control, four animals from G-NcSpain7 and four animals from G-NcSpain1H were culled at 10 dpi, while three animals from G-Control, five animals from G-NcSpain7 and five animals from G-NcSpain1H were culled at 20 dpi.

### Parasites

Nc-Spain7 and Nc-Spain1H tachyzoites were routinely maintained in cultured MARC-145 cells, and *inoculum* was prepared as described previously [21]. The same limited parasite passage numbers for both isolates were used for the experimental infection [11] to ensure the maintenance of their in vivo biological characteristics and avoid adaptation to the host cell [22]. Briefly, tachyzoites were recovered from culture flasks when they were still largely intracellular, and at least 80% of the parasitophorous vacuoles were undisrupted. Tachyzoite numbers were determined by Trypan blue exclusion followed by counting in a Neubauer chamber, and parasites were resuspended in PBS at the required dose of 10^7^ tachyzoites in a final volume of 2 mL. Tachyzoites were administered to heifers within 1 h of harvesting from tissue culture.

### Clinical monitoring and sampling

Cattle were observed daily before and after inoculation throughout the entire experimental period. Rectal temperatures were recorded daily from 6 days prior to challenge to 14 dpi and weekly from 14 dpi onward. Animals with temperatures above 39.5 °C were considered to be febrile. Foetal viability was checked once a week by ultrasound scanning of foetal heartbeat and movements. Blood samples were collected by coccygeal venipuncture at days −6 and −1 and twice a week until the end of the experiment for further analyses.

Animals were sedated with xylazine hydrochloride (Rompun; Bayer, Mannheim, Germany) and euthanised by an IV overdose of embutramide and mebezonio iodide (T61; Intervet, Salamanca, Spain). Post-mortem examination of the heifers was performed immediately after euthanasia. Foetuses were separated from the placenta, and 18 placentomes (6 cranial, medial and caudal) were randomly recovered from each placenta. Half of the placentomes from each area were carefully detached by hand, and maternal caruncles (CA) and foetal cotyledons (CO) were separated. Full placentomes were transversally cut in slices measuring 2–3 mm in thickness, which were distributed for storage in 10% formalin (Sigma-Aldrich, Saint Louis, MO, USA) for histopathological examinations. Both full placentomes and CA and CO sections were also stored at −80 °C for parasite DNA detection by PCR. Foetal tissues included the brain (FB), heart, liver (FL), lung and a portion of semitendinosus skeletal muscle, which were maintained at −80 °C for DNA extraction and fixated in 10% formalin. Blood and foetal thoracic and abdominal fluids were also collected when possible and maintained at −80 °C for serological analysis. Heifer tissues, including pre-scapular and ileofemoral lymph nodes, were also collected for PCR and histopathological analysis.

### Histopathology and lesion quantification

After fixation for 2 weeks, maternal and foetal samples and placentomes were trimmed and conventionally processed for embedding in paraffin wax and haematoxylin and eosin (HE) staining. Histological slides were studied under an optical microscope. Lesion quantification at placentome samples was performed through a computer-assisted morphometric analysis in HE stained sections following a previously described procedure [23]. Among the parameters evaluated were the number and size of necrotic foci (NF and ASF), as well as the total area of necrotic lesions (%LES) affecting the interdigitate area of the placentome. In addition, the accumulation of proteinaceous material (eosinophilic) and cellular debris in the haemophagus subchondral area of the placentome was also measured, and the results are expressed as a ratio between the area occupied by the proteinaceous exudate in the haemophagus zone and the total area of the placentome.

### Tissue DNA extraction and PCR determinations

DNA extraction and PCR determinations were carried out as described elsewhere [7, 23]. Briefly, genomic DNA was extracted from 20 to 100 mg of maternal and foetal tissue samples using the Maxwell^®^ 16 Mouse Tail DNA Purification Kit (Promega, Wisconsin, USA). Parasite DNA was detected by nested PCR adapted to a single tube from the internal transcribed spacer (ITS1) region of *N. caninum* using TgNN1-TgNN2 and NP1-NP-2 as external and internal primers, respectively [7, 24, 25]. DNA quantification was performed by real-time PCR using the equipment ABI 7500 FAST (Applied Biosystems, Foster City, CA, USA) and targeting Nc-5 as described previously [26]. Detailed information concerning DNA extraction and PCR is given in Additional file 1.

### IFN-γ responses in sera

IFN-γ levels in sera from dams were measured by the Bovine IFN-γ ELISA development kit (Mabtech AB, Sweden) following the manufacturer’s recommendations. The colour reaction was developed by the addition of 3,3′,5,5′-tetramethylbenzidine substrate (TMB, Sigma-Aldrich, Spain) and incubated for 5–10 min in the dark. Reactions were stopped by adding 2N H_2_SO_4_ when the first point of the standard curve reached a DO of 0.7 at 620 nm. Then, plates were read at 450 nm. The cytokine concentrations were calculated by interpolation from a standard curve generated with recombinant cytokines provided with the Bovine IFN-γ ELISA development kit (Mabtech AB, Sweden).

### *N. caninum*-specific IgG responses

*Neospora*-specific IgG antibody levels were measured in maternal serum by ELISA [7]. IgG1 and IgG2 subclasses were also assessed by ELISA using peroxidase-conjugated sheep anti-bovine IgG1 and IgG2 antibodies (Serotec, Oxford, UK) at 1:1000 as secondary conjugates. For each plate, the OD values were converted into a relative index percent (RIPC) using the following formula: RIPC = (OD_405_ sample − OD_405_ negative control)/(OD_405_ positive control − OD_405_ negative control) × 100. A RIPC value ≥ 12 indicates a positive result.

Indirect fluorescent antibody test (IFAT) and Western blotting (WB) were carried out to detect specific IgG anti-*Neospora* antibodies in foetal blood and foetal thoracic and abdominal fluids. IFAT was carried out following the methodology previously described [27]. Samples were diluted at two-fold serial dilutions in PBS starting at 1:8 up to the end point titre. Intact tachyzoite membrane fluorescence at a titre ≥ 8 was considered a positive reaction. WB was carried out as described previously [28]. After blocking overnight, the membranes containing tachyzoite extracts were incubated with foetal sera and fluids diluted 1:20 and incubated for 1.5 h at room temperature. After washing, the membranes were incubated with 1:1200 peroxidase-conjugated monoclonal goat anti-bovine IgG (Thermo Fisher Scientific, Waltham, MA, USA) for 1 h, washed and developed using 4-chloro-1-naphtol (Bio-Rad Laboratories, CA, USA) as a substrate.

### Statistical analysis

Differences in PCR detection of parasite DNA in maternal, foetal and placental tissues were evaluated using *χ*^2^ or Fisher’s exact F-test. Parasite burdens were analysed using the non-parametric Mann–Whitney U test. Occurrence of foetal death was analysed by the Kaplan–Meier survival method to estimate the percentage of viable foetuses (VF) at each time point [29]. The foetal survival curves of the infected groups were then compared with the Gehan–Wilcoxon test. Differences in histological scoring were analysed using the non-parametric Kruskal–Wallis test followed by Dunn’s test for all pairwise comparisons. Finally, a two-way ANOVA test followed by a Tukey’s multiple comparisons test, was performed to compare rectal temperatures, antibody responses and IFN-γ kinetics in sera.

Statistical significance for all analyses was established with *P* < 0.05. All statistical analyses were carried out using GraphPad Prism 5 v.5.01 software (San Diego, CA, USA).

## Results

A summary of results (clinical outcome, lesions, parasite distribution and IgG responses) in heifers and foetuses inoculated with PBS, 10^7^ tachyzoites of Nc-Spain7 isolate or Nc-Spain1H isolate at 110 dg and culled at 10 or at 20 dpi is shown in Table 1.

**Table 1:** **Summary of early infection dynamics in heifers and foetuses from G-Control, G-NcSpain7 and G-NcSpain1H**

Culling date	*Inoculum*	Ear tag	Pregnancy outcome	*Placenta*		*Foetus*			*N. caninum*-specific IgG levels in dams
				Histopathology	DNA detection	Histopathology	DNA detection	*N. caninum*-specific IgG	
10 dpi	*PBS*	5702	L	PA*	−	−	−	No	No
		1334	L	PA*	−	−	−	No	No
		6676	L	PA*	−	−	−	No	No
	*Nc*-*Spain7*	1600	L	PA**	−	−	−	No	No
		9665	L	FN*, PA**	CA++, CO+	−	Li+	No	No
		5850	L	PA**	−	−	−	No	No
		9661	L	PA**	−	−	Li++	No	No
	*Nc*-*Spain1H*	9131	L	PA**	−	−	−	No	No
		3712	L	PA**	−	−	−	No	No
		5925	L	PA**	−	−	−	No	No
		9671	L	PA**	−	−	−	No	No
20 dpi	*PBS*	3710	L	PA*	−	−	−	No	No
		6671	L	PA*	−	−	−	No	No
		6377	L	PA*	−	−	−	No	No
	*Nc*-*Spain7*	3581	D	FN***, PA***	CA+++, CO+++	Li**, Lu**, H, Sk, CNS^†^	CNS++	No	Yes (13 dpi)
		7934	D	FN**, PA***	CA+++, CO+++	Li**, Lu***, H, Sk, CNS^†^	CNS++, Li+++	No	Yes (9 dpi)
		7992	L	FN**, PA***	CA+++, CO+++	Li*, Lu*, H, Sk, CNS	CNS++	No	Yes (13 dpi)
		4405	L	FN**, PA***	CA+++, CO+++	Li*, Lu*, H, Sk, CNS	CNS+++	No	Yes (13 dpi)
		5082	L	FN***, PA***	CA+++, CO+++	Li*, Lu*, H, Sk, CNS	CNS+++	No	Yes (16 dpi)
	*Nc*-*Spain1H*	7725	L	PA**	−	−	−	No	No
		9677	L	PA**	−	−	−	No	Yes (13 dpi)
		7649	L	PA**	−	−	−	No	Yes (16 dpi)
		9638	L	PA**	CA+, CO ++	−	−	No	Yes (16 dpi)
		3894	L	PA**	−	−	−	No	No

PA*, **, *** (protein accumulation): arbitrary degree of accumulation of sera (eosinophilic) and cellular debris at the haemophagus subchondral area of the placentome.

FN*, **, *** (focal necrosis): arbitrary degree of focal necrosis with inflammatory infiltrate in the interdigitate zone of the placentome.

Li*, ** (liver): perivascular aggregation of lymphocytes, macrophages and plasma cells and mild multifocal necrotic foci.

Lu*, **, *** (lung): aggregation of mononuclear cells in the parenchyma, perivascular mononuclear inflammation and mild multifocal necrosis.

CNS^†^: CNS autolytic, evaluation of lesions was not possible.

CA/CO+, ++, +++ (caruncle/cotyledon): 1–3 positive samples, 4–6 positive samples or 7–9 positive samples.

Li/CNS+, ++, +++ (liver/central nervous system): 1, 2 or 3 positive samples.

−: negative/no lesion.

L: live foetus, D: dead foetus, Sk: skeletal muscle, H: heart, CNS: central nervous system.

### Clinical observations

**Figure 1:**
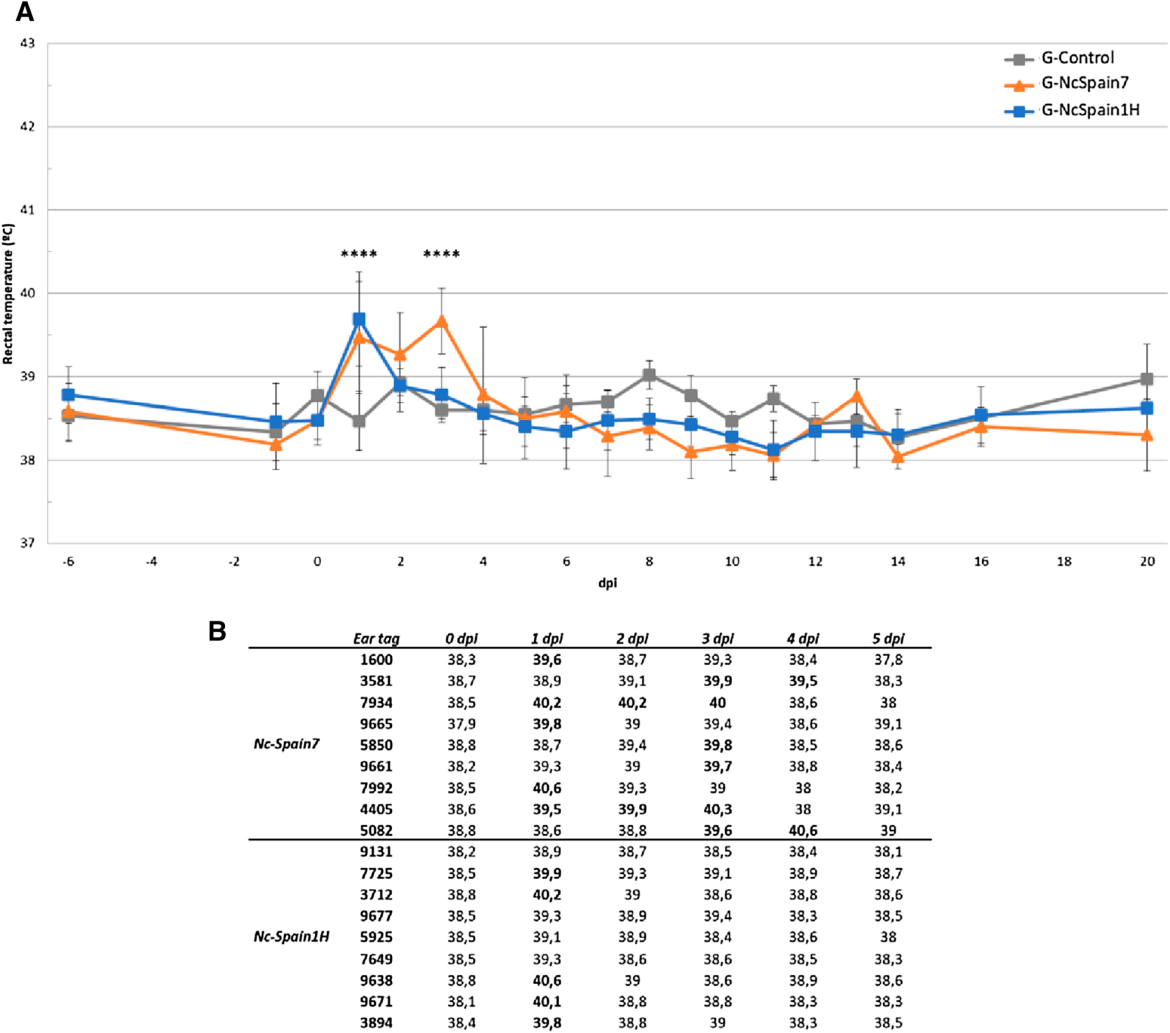
**Rectal temperatures.** The mean rectal temperatures of animals from G-Control, G-NcSpain7 and G-NcSpain1H during the experiment are represented in the graphic (**A**). The exact temperatures of each infected animal recorded during the first 5 dpi are represented in table (**B**). **** Indicates *P* < 0.0001 significant differences.

The mean rectal temperatures of animals from G-Control, G-NcSpain7 and G-NcSpain1H are represented in Figure 1. Five animals from G-NcSpain7 and 5 animals from G-NcSpain1H exhibited fever at 1 dpi. Six animals from G-NcSpain7 also exhibited fever at 3 dpi, and two of these animals maintained fever until 4 dpi. The mean rectal temperatures of G-NcSpain7 increased significantly (> 39.5 °C) at 1 and 3 dpi, and the mean rectal temperature of G-NcSpain1H only increased significantly at 1 dpi compared to the uninfected G-Control group (*P* < 0.0001; two-way ANOVA test). Significant differences between infected groups were found at 3 dpi when a second peak of fever was detected in G-NcSpain7 but not in G-NcSpain1H (*P* < 0.0001; two-way ANOVA test). Interestingly, only 5 out of 9 G-Nc-Spain1H heifers presented fever, whereas all G-Nc-Spain7 heifers were febrile at some time during the experimental period. Rectal temperatures of G-Control animals remained below 39 °C.

Foetal mortality was not detected until 20 dpi. Foetal death was detected during culling in two heifers (3581 and 7934) from G-NcSpain7 as detailed below. However, foetuses from G-Control and G-NcSpain1H remained viable throughout the experiment. The comparative analysis of foetal survival curves between infected groups showed non-significant differences (*P* = 0.13; Gehan–Wilcoxon test).

### Pathology and lesion quantification

#### Gross lesions

Placental detachment from the uterus, and autolyzed CO, were found in the two heifers (3581 and 7934) from G-NcSpain7 that were culled at 20 dpi and presented foetal mortality. In these cases, foetuses were swollen because of subcutaneous oedema and showed a degree of autolysis. Apart from these findings, no evident gross lesions were found in the placentas, foetuses or maternal lymph nodes studied from any of the other heifers.

#### Microscopic lesions

Maternal lymph nodes: histological changes were not found in any lymph node.

Placentomes: two different histological changes were found in the placentomes.

The first change consisted of focal necrosis with a variable degree of inflammatory infiltrate adjacent to the lesion, randomly distributed within the interdigitate zone of the placentome (Additional files 2A and B). This lesion was only found in G-NcSpain7 heifers, but there were differences within this group, as only one heifer culled at 10 dpi (9665) showed this lesion, affecting only one out of nine studied placentomes. The lesion was also found in all G-Nc-Spain7 animals culled at 20 dpi. All the parameters quantified in these lesions, NF, ASF and %LES, were higher in animals culled at 20 dpi than those found in the only animal with placental lesions culled at 10 dpi. Among those culled at 20 dpi, one of them (3581, non-viable foetuses-NVF-) had more NF and more %LES than the rest (*P* < 0.01; Kruskal–Wallis test) (Additional files 3A and C). The ASF was higher in two animals (3581, NVF and 5082, VF) than in the other three (Additional file 3B). When studying the influence of the location of the placentomes (cranial, medial or caudal) on the evaluated parameters, no differences were found between the five heifers culled at 20 dpi. However, when analysing those two animals with higher ASF, medial and caudal CO showed higher %LES (*P* < 0.05; Kruskal–Wallis test) than the cranial ones (Additional file 3C).

**Figure 2:**
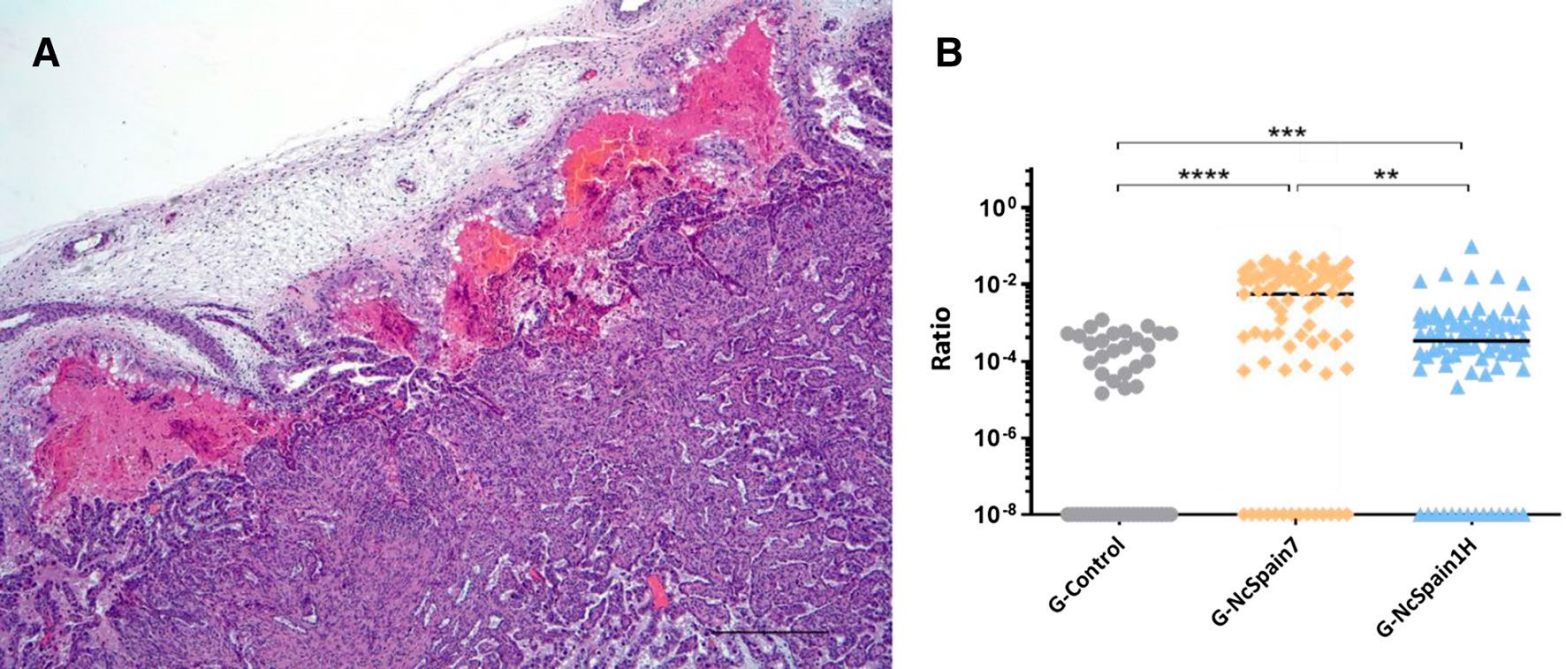
**Proteinaceous exudate at the haemophagus area of the placentome.** Representative image of accumulation of proteinaceous material and cellular debris at the haemophagus subchondral area of a placentome from G-NcSpain1H at 20 dpi. HE. ×2 (**A**), and dot-plot graph showing significant differences between groups (**B**). Each dot represents individual values of relative area occupied by the exudate in each placentome analysed, and medians are represented as horizontal lines. ****, *** and ** indicate *P* < 0.0001, *P* < 0.001 and *P* < 0.01 significant differences. Bar 1000 µm.

The second histological change found in placentomes was the accumulation of proteinaceous material (eosinophilic) and cellular debris at the haemophagus area of the placentome, i.e., extravasated plasma (Figure 2A). This accumulation was found in all the animals from the study, including G-Control. However, comparing the amount of extravasated plasma, measured as the relative area occupied by the proteinaceous material in the haemophagus area, there were clear differences between groups (Figure 2B). Placentomes from G-Control showed less accumulation than G-NcSpain7 (*P* < 0.0001; Kruskal–Wallis test) and G-NcSpain1H (*P* < 0.001; Kruskal–Wallis test), but the accumulation in G-NcSpain7 was higher than in G-NcSpain1H (*P* < 0.05; Mann–Whitney U test). Comparing the differences within each group depending on the day of culling (10 vs 20 dpi), differences were found only at G-NcSpain7, where animals culled at 10 dpi showed less accumulation than those culled at 20 dpi.

Foetal viscera. Only the five foetuses from G-NcSpain7 heifers culled at 20 dpi showed histological lesions. The livers of all five foetuses showed perivascular aggregation of lymphocytes, macrophages and plasma cells. NVF (3581 and 7934) also showed mild multifocal necrotic foci in the liver with scant presence of inflammatory cells related to them. In addition, all foetuses from G-NcSpain7 culled at 20 dpi showed scant, randomly distributed aggregation of mononuclear cells in the lung parenchyma. NVF (3581 and 7934) also showed similar lesions in the lungs plus perivascular infiltration of mononuclear cells and, in one of them (7934), mild multifocal necrosis. Finally, we also found mild mononuclear myositis and myocarditis (Additional file 2C) in all five foetuses. Multifocal randomly distributed small aggregation of mononuclear cells in the neuropil of the brain (Additional file 2D) was observed only in VF as the brain samples from NVF were too autolytic to allow proper histological evaluation.

### Parasite distribution and burden in placental and foetal tissues

**Figure 3:**
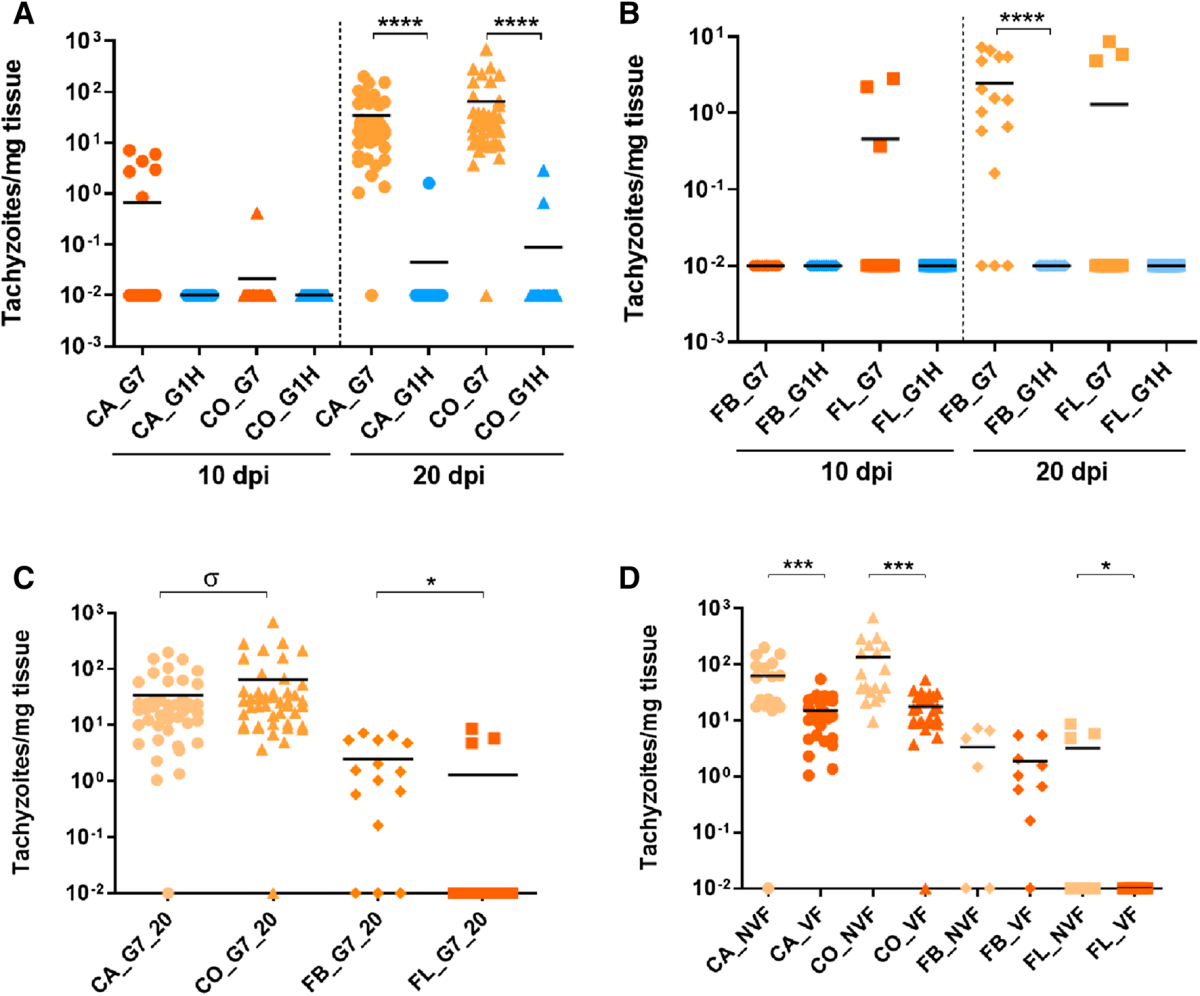
***N. caninum*** **burdens in placental and foetal tissues.** Comparative of parasite burdens quantified by qPCR in nested-PCR positive samples from CA and CO (**A**) and FB and FL (**B**) from G-NcSpain7 and G-NcSpain1H heifers culled at 10 and 20 dpi. **C** Comparative of parasite quantification by qPCR between CA and CO and between FB and FL from G-NcSpain7 heifers culled at 20 dpi. **D** Comparative of parasite quantification by qPCR in samples from CA, CO, FB and FL from NVF and VF foetuses from G-NcSpain7 culled 20 dpi. Each dot represents individual values of parasite burden, and medians are represented as horizontal lines. The *N. caninum* detection limit by real-time PCR was 0.1 parasites, and negative samples (0 parasites) were represented on the log scale as < 0.1 (i.e., 10^−2^). ****, ***, and * indicate *P* < 0.0001, *P* < 0.001 and *P* < 0.05 significant differences. *σ* indicates *P* < 0.1 tendency towards significant differences.

Parasite burdens are represented in Figure 3.

#### Maternal tissues

*Neospora caninum* DNA was only detected in 1 out of 15 pre-scapular lymph node samples in one heifer from G-NcSpain1H culled at 20 dpi (9638).

#### Placental tissues

In G-NcSpain7, *Neospora* DNA was detected sporadically in CA (4/36) and CO (1/36) samples belonging to one animal culled at 10 dpi (9665) and in 44 out of 45 CA and 44 out of 45 CO samples of animals culled at 20 dpi. The differences in the frequency of parasite detection between animals from G-NcSpain7 culled at 10 and at 20 dpi were statistically significant for CA and for CO (*P* < 0.0001; Fisher exact test). In G-NcSpain1H, all CA and CO samples from animals culled at 10 dpi were negative, and only 1 out of 45 CA and 4 out of 45 CO samples from one animal culled at 20 dpi (9638) were *N. caninum* DNA positive. The frequency of detection in CA and CO in G-NcSpain7 was significantly higher than in G-NcSpain1H culled at 20 dpi (*P* < 0.0001; Fisher exact test). Placental samples from G-Control animals were negative.

The parasite burden in CA and CO, measured as the number of tachyzoites per mg of tissue, was analysed in *N. caninum* DNA-positive samples. Higher parasite burdens were found in CA and CO from G-NcSpain7 at 20 dpi than in samples from G-NcSpain7 at 10 dpi (*P* < 0.0001; Mann–Whitney U test). Slightly higher parasite burdens were found in CO than in CA samples from G-NcSpain7 culled at 20 dpi, although the differences were not statistically significant (*P* > 0.05; Mann–Whitney U test) (Figure 3C). The parasite burden was higher in CA (*P* ≤ 0.001; Mann–Whitney U test) and CO (*P* < 0.0001; Mann–Whitney U test) from animals with NVF (3581 and 7934) than those carrying VF (7992, 4405 and 5082) of G-NcSpain7 at 20 dpi (Figure 3D). In contrast, differences in the parasite burden in CA and CO between animals from G-NcSpain1H culled at 10 and 20 dpi were not found (*P* > 0.5; Mann–Whitney U test). Comparing animals from infected groups culled at 20 dpi, higher parasite burdens in CA and CO were detected in G-NcSpain7 than in G-NcSpain1H (*P* < 0.0001; Mann–Whitney U test) (Figure 3A).

#### Foetal tissues

Regarding foetal tissues, 12 out of 15 FB samples from G-NcSpain7 foetuses at 20 dpi were positive by PCR, whereas all FB samples from G-NcSpain7 foetuses at 10 dpi were negative. All FBs from G-NcSpain1H foetuses culled at 10 or 20 dpi were negative. On the other hand, 2 G-NcSpain7 foetuses at 10 dpi (3/12) and one G-NcSpain7 foetus at 20 dpi (3/15) presented *N. caninum* positive FL samples, although differences between culling at 10 or 20 dpi were not found (*P* = 1; Fisher exact test). FL from all G-NcSpain1H foetuses were negative for *N. caninum* DNA detection. FB and FL samples from G-Control were negative.

Higher parasite burdens were found in FB samples from G-NcSpain7 culled at 20 dpi than at 10 dpi (*P* < 0.0001; Mann–Whitney U test), but differences were not found in FL (*P* > 0.5; Mann–Whitney U test) (Figure 3B). In addition, a higher parasite burden was found in FB than in FL in G-NcSpain7 at 20 dpi (Figure 3C). Comparing animals carrying VF and NVF from G-NcSpain7 at 20 dpi, higher parasite burdens were found in the FL of NVF (*P* < 0.05; Mann–Whitney U test), whereas no differences were found in the FB (*P* > 0.5; Mann–Whitney U test) (Figure 3D).

### IFN-γ kinetics in sera

**Figure 4:**
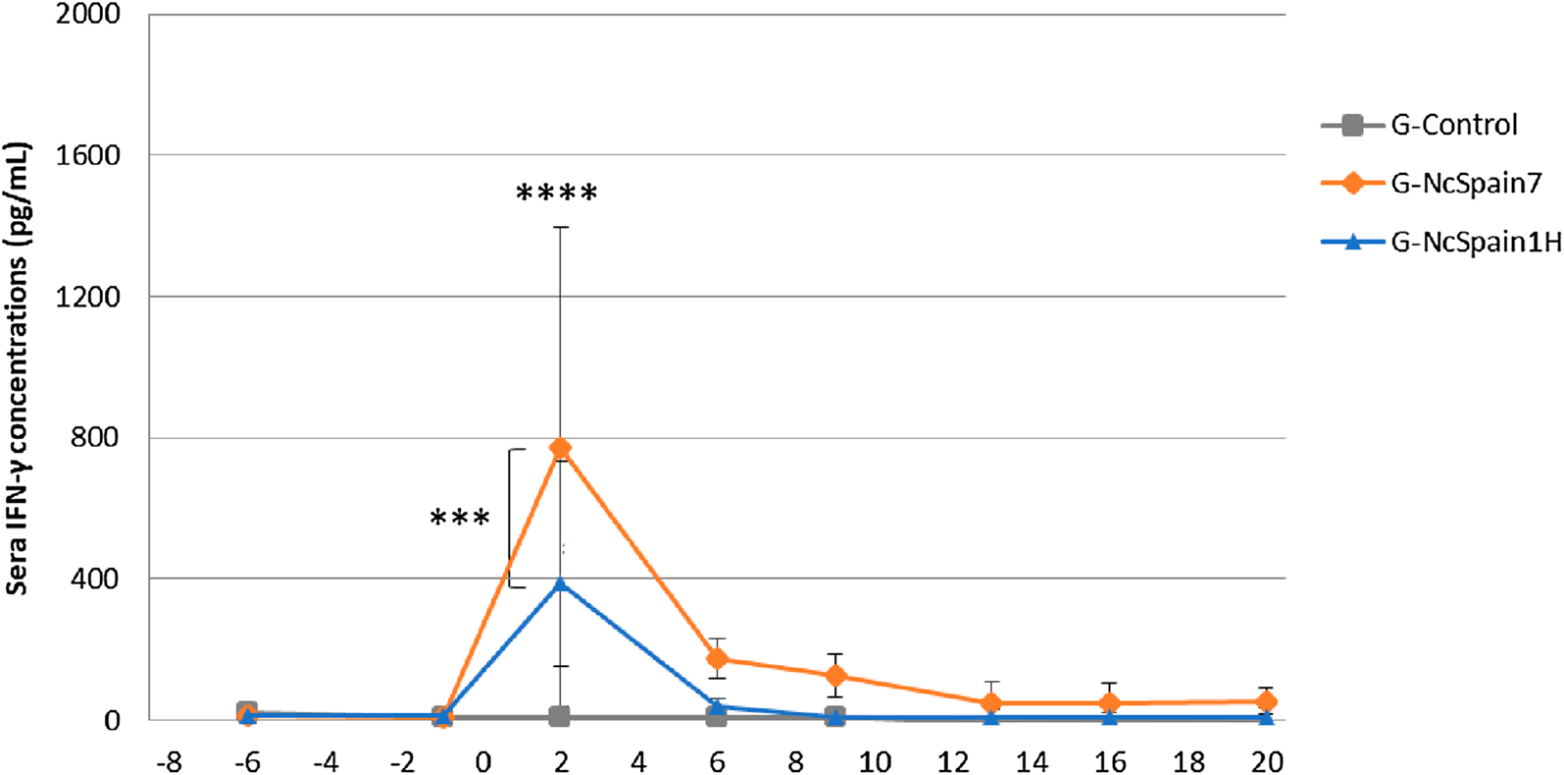
**IFN-γ kinetics in sera.** Sera concentration of IFN-γ determined by ELISA in G-Control, G-NcSpain7 and G-NcSpain1H. **** and *** indicate *P* < 0.0001 and *P* < 0.001 significant differences.

A peak of IFN-γ production was detected at 2 dpi in both infected groups with respect to the control group G-Control (*P* < 0.0001 and *P* = 0.0013 in G-NcSpain7 and G-NcSpain1H, respectively; two-way ANOVA test). All animals from G-NcSpain7 and G-NcSpain1H presented increased levels of IFN-γ at 2 dpi. Differences between infected groups were also found, with the increase of IFN-γ being higher in Nc-Spain7-infected animals than in Nc-Spain1H-infected animals (*P* < 0.0002; two-way ANOVA test) (Figure 4).

### Specific anti-*Neospora* IgG responses in heifers and foetuses

**Figure 5:**
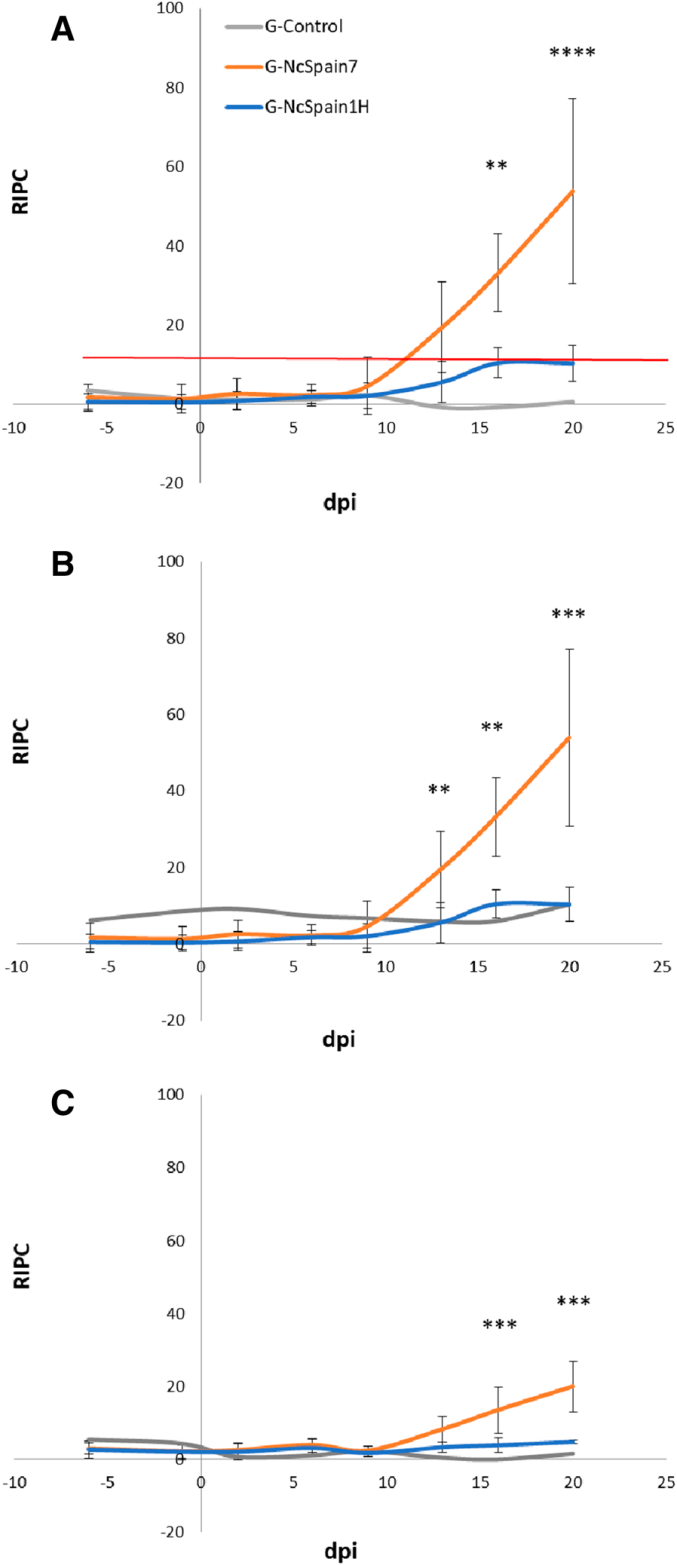
***N. caninum*-specific humoural immune responses.** Serum levels of total IgG (**A**), IgG1 (**B**) and IgG2 (**C**) antibodies against *N. caninum* in G-Control, G-NcSpain7 and G-NcSpain1H. Immunoglobulin levels are expressed as a relative index percent (RIPC) according to RIPC = (OD_405_ sample − OD_405_ negative control)/(OD_405_ positive control − OD_405_ negative control) × 100. Each point represents the mean + SD at different sampling times. The red line indicates the ELISA cut-off point from which samples are considered positive. **** and ** indicate *P* < 0.0001 and *P* < 0.001 significant differences.

*Neospora caninum*-specific antibody responses (total IgG, IgG1 and IgG2) are shown in Figure 5. An earlier detection of *N. caninum* antibodies was observed in G-NcSpain7 (9 dpi) (7934) than in G-NcSpain1H (13 dpi). All animals from G-NcSpain7 seroconverted from 13 dpi, while only 3 out of 5 animals from G-NcSpain1H seroconverted between 13 (9677) and 16 dpi (7649 and 9638). Total IgG levels were significantly higher from 13 dpi until the end of the experiment in G-NcSpain7 compared to G-Control (*P* < 0.05; two-way ANOVA test). No significant increase in the antibody levels was found in G-NcSpain1H during the experimental period compared to the control group (*P* > 0.05; two-way ANOVA test). Total IgG levels of G-NcSpain7 were higher than G-NcSpain1H at 16 dpi (*P* < 0.05; two-way ANOVA test) and at 20 dpi (*P* < 0.0001; two-way ANOVA test). No significant differences were found in G-NcSpain7 at 20 dpi between animals carrying NVF (3581 and 7934) and VF (7992, 4405 and 5082) (Figure 5A).

IgG1 and IgG2 kinetics were similar to those observed with total IgG levels. Higher levels of IgG1 and IgG2 were found in G-NcSpain7 than in G-Control at 16 and 20 dpi (*P* < 0.01; two-way ANOVA test). There were no significant differences in IgG1 and IgG2 levels between G-NcSpain1H and G-Control. Comparing both infected groups, higher IgG1 and IgG2 levels were found in G-NcSpain7 than in G-NcSpain1H at 16 and 20 dpi (*P* < 0.01; two-way ANOVA test), and higher IgG1 levels were also found in G-NcSpain7 than in G-NcSpain1H at 13 dpi (*P* < 0.01; two-way ANOVA test). No significant differences in IgG1 and IgG2 were found between animals carrying NVF (3581 and 7934) and VF (7992, 4405 and 5082) in G-NcSpain7 at 20 dpi (Figures 5B and C).

*Neospora*-specific IgG was not detected in foetal serum or foetal fluids by IFAT and WB.

## Discussion

Abortion due to *N. caninum* infection appears more frequently at mid-gestation in naturally infected cattle. However, few studies of pregnant bovine models of neosporosis at this gestation time have been reported, especially those investigating early infection dynamics. In the present work comparisons between high (Nc-Spain7)- and low (Nc-Spain1H)-virulence isolates of *N. caninum* inoculated at mid-gestation were done at early time points post-infection (10 and 20 dpi). The results from this experimental model will increase the knowledge about biological differences found between high- and low-virulence isolates in vivo, clarifying some of the key events involved in the pathogenesis of bovine neosporosis.

Half of the animals from both infected groups showed fever as the first clinical sign associated with *N. caninum* infection, which is in agreement with previous reports where a transient rise in body temperature was recorded during the first week post-infection, likely the consequence of the first cycle of parasite replication in host tissues [6, 7, 11, 12, 17, 24, 30]. A second peak of fever was detected at 3 dpi only in Nc-Spain7-infected animals, which suggests an earlier and higher replication of this isolate, leading to a second antigenic exposition of Nc-Spain7 tachyzoites. Similarly, high doses of the Nc-1 isolate were associated with a bi-phasic increase in rectal temperature [15]. Moreover, all G-NcSpain7-heifers were febrile, whereas only half of G-NcSpain1H animals showed fever. Differences between isolates may be explained by a more efficient replication of Nc-Spain7, as was previously demonstrated in vitro [31–33] and in vivo [21].

Peripheral immune responses, both cellular and humoural, were assessed in dams’ sera during the experiment. IFN-γ was detected in both infected groups at 2 dpi, demonstrating that *N. caninum* tachyzoites activated the innate immune response, which is crucial for host defence against intracellular pathogens [34–36]. Specific antibodies against *N. caninum* were detected in all Nc-Spain7 heifers slightly earlier than in previous works [9, 11, 16], whereas later seroconversion, and in fewer animals, was found in three Nc-Spain1H-infected heifers. Previous reports suggested that Nc-Spain7 may be able to induce a higher antibody response, whereas antigenic stimulation seems to be more reduced in Nc-Spain1H [7, 18, 37]. It is unknown if the immune response developed in Nc-Spain 1H-infected animals was able to reduce parasite burden, limiting the tissue damage or if the low capacity of the isolate to multiply in host tissues may be associated with the reduction or absence of repeated antigenic stimulus [18, 38]. Therefore, infections with low-proliferative isolates of *N. caninum*, as Nc-Spain1H would be detected later in the field, in fact IgG was detected between 18 and 88 days post-challenge (dpc) with the peak around 50 dpc [37], or even not detected if abortion or clinical signs are not present, although good control measures as resampling of animals would avoid possible diagnostic inconvenience.

In this study, culling as early as 10 and 20 dpi was effective in showing clear differences between isolates of variable virulence. At 10 dpi, few placental samples from one Nc-Spain7-infected animal were positive for *N. caninum,* and one of them showed focal necrosis, demonstrating the colonization of the placenta by this isolate. Early detection of Nc-Spain7 at 10 dpi may be associated with its higher abilities for invasion and proliferation in placental cells. To the best of our knowledge, none of the previous experiments studied the dynamics of the infection as early as 10 dpi, however similar to our observations, focal necrosis was described in placentomes at 14 dpi with the Nc-1 isolate [15] and at 2 wpi with the Nc-Spain7 isolate [11]. In addition to focal necrosis, differences in the plasma extravasation were found between infected and control animals. The extravasation of erythrocytes and plasma into the haemophagus zone of the placentome is a normal finding in healthy animals [39]. However, in this study, higher extravasation was found in infected animals at 10 and 20 dpi. Previous studies have described serum leakage in relation to necrotic and inflammatory foci in the interdigitate area of the placenta [9, 15], but the increase in the proteinaceous material in the haemophagus area has not been previously reported. This increase may appear as one of the initial changes in the placenta associated with *N. caninum* infection, and it is tempting to hypothesize that it might be related to changes in vascular permeability. It has been recently shown that complement and coagulation cascades were modified after *N. caninum* infection in trophoblast cells in vitro [40]; therefore, studying early vascular events in the placenta after *N. caninum* infection could be an interesting future research. *N. caninum* was also detected in the liver of two Nc-Spain7-infected foetuses, indicating that the high-virulence isolate is already transmitted to the foetus at 10 dpi. Previous studies have suggested the crossing and presence of tachyzoites in foetal tissues as early as 10 dpi [15, 41]. Our findings suggest that the liver is the first target organ in the foetus, which most likely represents the gateway for the parasite to invade the foetus through the umbilical vein, replicating in the parenchyma and spreading through the foetal body, as previously observed in sheep [23]. However, antibodies against *N. caninum* were not found in serum or corporal fluids from any foetus, probably because at least 6 weeks between maternal infection and foetal seroconversion are needed [11, 17].

In contrast, Nc-Spain1H was not found in any placental or foetal tissue, and specific humoural responses were not found either in Nc-Spain1H-infected foetuses. Lesions were not found either in placentas or in foetuses from G-NcSpain1H, apart from serum extravasation in placentomes. However, IV inoculation is supposed to disseminate the parasite quickly through the organism. We hypothesize that the presence of Nc-Spain1H in the placenta at 10 dpi is very low, since we were unable to detect the parasite, which is associated with lower invasion and replication abilities, as previously observed in placental tissues in vitro, especially in caruncular cells [32]. In addition, a higher stimulation of the innate immune responses by the low-virulence isolate at the placental level was suggested at early time points post-infection in vitro [42], which could explain the more effective control of the parasite, thereby contributing to its lower proliferation.

At 20 dpi, foetal death was detected in two Nc-Spain7-infected heifers, whereas it was not detected in any Nc-Spain1H-infected heifer. All G-NcSpain7 animals presented almost 100% positive placental samples, which is in keeping with previous studies where Nc-Spain7 or other isolates showed dissemination in placental tissues early after infection (2–4 wpi) when inoculation was carried out at mid-gestation (110–140 dg) [9, 11, 15, 37]. Our results again demonstrated the “tropism” of *N. caninum* for the bovine placental tissue, which seems to be one of the most appropriate niches for its multiplication. Placental necrosis was observed in G-NcSpain7 animals at 20 dpi associated with high parasite burdens. The extravasation of proteinaceous material and cellular debris in the haemophagus area in G-NcSpain7 was larger at 20 dpi than at 10 dpi and larger than in G-NcSpain1H animals, suggesting a correlation between the presence and severity of this histological change and infection by *N. caninum*, since larger areas of extravasation were observed in animals with higher parasite burden and infected with more virulent isolates. Parasite was found in most FB samples from G-NcSpain7, and inflammatory infiltrate and lesions compatible with *N. caninum* were found especially in foetal CNS, which is in keeping with previous observations where the brain was defined as a target tissue for *N. caninum* [9, 11, 43] but also in lung, skeletal muscle, heart and liver. Inflammatory infiltrate in foetal organs supports the hypothesis that at least partial foetal immunocompetence is already developed at this time, although no specific antibodies were found in foetal sera or in foetal fluids, as explained above.

In contrast to those results observed after infection with the isolate of high-virulence Nc-Spain7, the infection with the low-virulence isolate Nc-Spain1H did not induce foetal death, and only one G-NcSpain1H animal presented positive placental samples at 20 dpi, similar to a previous experimental study at early gestation [18]. In addition, there were no evident lesions at the placenta. Taken together, these results suggest a limited colonization of maternal placenta by Nc-Spain1H, which is consistent with the low proliferation rate of this isolate under in vitro conditions [31, 32]. In addition, four CO samples were positive, suggesting that as demonstrated in vitro in F3 cells, the foetal compartment of the placenta may be the target cell and the preferential niche for parasite multiplication, whereas caruncular cells seem to play a barrier role for the placenta, limiting the invasion and multiplication of the parasite [32]. Moreover, higher activation of the innate immune responses, specifically TLR-2, on the maternal side as observed in vitro [42], may contribute to the elimination of the tachyzoites, diminishing the burden in the caruncle and limiting tissue damage. Despite the absence of parasite DNA, lesions or foetal antibodies in G-NcSpain1H foetuses at 20 dpi, the identification of parasite DNA on CO indicated the transmission of this isolate to the foetal compartment. In fact, the origin of this isolate (from a dairy herd with high intra-herd *N. caninum* seroprevalence) [44] and a previous experimental infection at early gestation [18] also corroborate that transmission of Nc-Spain1H to the foetus does occur. It is therefore tempting to hypothesize that if the experimental design of the study had allowed a longer gestation, the parasite might have been transmitted to the foetus.

Related to the pathogenesis of abortion, in the present work, higher parasite burdens and more severe lesions were detected in placentomes from one animal carrying NVF (3581) compared to VF, demonstrating that replication of the parasite at the maternal-foetal interface may be an important factor of foetal mortality [45]. On the other hand, resolution of placental lesions was demonstrated at 42 dpi [15], which indicated that progression of infection had been halted by the dam and the foetus and could be a reversible process in some cases. Moreover, our results showed that the %LES of the placenta was low, lesions showed a focal distribution and severity of the lesions did not seem sufficient to justify the foetal death by themselves because hypoxia signs were not found in NVF and placental functions did not seem to be compromised. There were no differences in the parasite burden in FB between VF and NVF, and only slightly higher parasite burden in FL was found in NVF. In addition, similar lesions were found in the foetal brain, liver, lung, heart and skeletal muscle of all Nc-Spain7 foetuses at 20 dpi. Brain lesions could be evaluated only in VF since NVF presented autolysis of the CNS. A key question that remains unsolved is the role of the maternal and foetal immune responses in the outcome of the infection.

In summary, wider parasite dissemination with earlier transmission to the foetus and foetal death were found after infection with the high-virulent isolate Nc-Spain7 as soon as 10 and 20 dpi, respectively. All these findings seem to be related to a better capacity of this isolate to invade the placenta earlier and proliferate more efficiently. The pathogenesis of the abortion could not be determined with our findings, since placental and foetal burdens and lesions in VF and NVF would not explain by themselves the foetal death. Therefore, the roles of the maternal and foetal immune responses in the outcome of the infection should be investigated. However, this experiment was not designed to elucidate the cause of the abortion, and closer monitoring of the foetus and sequential sampling and culling are warranted in further research.

## Supplementary information

**Additional file 1. Materials and methods.** Description of the health and reproductive handling of the cattle and the tissue DNA extraction and PCR determinations.

**Additional file 2. Histological findings in placental and foetal samples.** (**A**) Placenta. G-NcSpain7 20 dpi. Three foci of necrotic placentitis with mild infiltration of inflammatory cells at the interdigitate area of the placentome HE. 4×. Bar 500 µm. (**B**) Placenta. G-NcSpain7 20 dpi. Focal necrosis with mild infiltration of inflammatory cells at the interdigitate area of the placentome. HE. 10×. Bar 200 µm. (**C**) Foetal heart. G-NcSpain7 20 dpi. Focal non-suppurative myocarditis. HE. 10×. Bar 200 µm. (**D**) Foetal brain. G-NcSpain7 20 dpi. Glia focus with a small area of necrosis at the centre of the lesions. HE. 10×. Bar 200 µm.

**Additional file 3. Quantification of necrosis foci (NF), size (ASF) and affected area (%LES) of these foci.** Graphs representing median number of cells, lower and upper quartiles (boxes) and minimum and maximum values (whiskers) of (**A**) number of NF in G-NcSpain7 culled at 20 dpi studied by individual animal or by placentome location, (**B**) ASF in G-NcSpain7 culled at 20 dpi studied by individual animal or by placentome location and (**C**) %LES in G-NcSpain7 culled at 20 dpi studied by individual animal, by placentome location or by placentome location in the placentomes of animals with higher ASF (3581 and 5082). ****, ***, ** and * symbols indicate *P* < 0.0001, *P* < 0.001, *P* < 0.01 and *P* < 0.05 significant differences.

## Abbreviations

F3: bovine placental trophoblast cell line
qPCR: Real-time Polymerase Chain Reaction
dpi: days post-infection
wpi: weeks post-infection
dg: days of gestation
dpc: days post-challenge
BVD: bovine viral diarrhea
PBS: phosphate buffered saline
ELISA: Enzyme-Linked ImmunoSorbent Assay
IV: intravenous
HE: haematoxylin–eosin
NF: necrotic foci
ASF: size of the necrotic foci
%LES: total area affected by necrosis
NVF: non-viable foetuses
VF: viable fetuses
CA: caruncle
CO: cotyledon
FB: foetal brain
FL: foetal liver
IFAT: indirect fluorescent antibody test
WB: Western blotting
