# Supplementary material for: Early Neospora caninum infection dynamics in cattle after inoculation at mid-gestation with high (Nc-Spain7)- or low (Nc-Spain1H)-virulence isolates
Source: Vet Res. 2019 Sep 24;50:72. doi: 10.1186/s13567-019-0691-6 (PMC6760050; doi:10.1186/s13567-019-0691-6)
Supplement: Supplementary file 1 — Additional file 1. Materials and methods. Description of the health and reproductive handling of the cattle and the tissue DNA extraction and PCR determinations. [file 13567_2019_691_MOESM1_ESM.docx]

**Health and reproductive handling of the cattle**

All animals were vaccinated against IBR virus and BVD virus with two doses of BOVILIS^®^ BOVIPAST RSP (MSD Animal Health, Milton Keynes, UK), separated for 4 weeks, and treated with antiparasitic products (Endoex® and Albendex®, s.p. veterinaria, s.a., Riudoms, Spain; Animec Plus®, Cenavisa laboratorios, Tarragona, Spain) following the manufacturer’s recommendations. Heifers were oestrus synchronised with the administration of: i) CIDR (Zoetis, New Jersey, USA) + 100 µg of synthetic gonadorelin analogue (Cystoreline^®^, CEVA, Barcelona, Spain) (day 0); ii) CIDR removing + 400 UI of equine serum gonadotropin (Folligon^®^, MSD Animal Health, Milton Keynes, UK) + 25 mg synthetic prostaglandin F2α analogue (Dinolytic^®^, Zoetis, New Jersey, USA) (day 5); iii) 25 mg synthetic prostaglandin F2α analogue (Dinolytic^®^, Zoetis, New Jersey, USA) (day 6); iv) 100 µg of synthetic gonadorelin analogue (Cystoreline^®^, CEVA, Barcelona, Spain) (day 7). Fifty-six hours after the first administration of PGF2α, two artificial insemination, 12 h apart, were carried out using semen from two Asturiana bulls seronegative to *N. caninum*. Pregnancy was confirmed by ultrasound scanning on day 35 after insemination, and twenty-four pregnant animals were selected for the experiment.

**Tissue DNA extraction and PCR determinations**

DNA extraction and PCR determinations were carried out as described elsewhere [7, 22]. Briefly, genomic DNA was extracted from 20-100 mg of maternal and foetal tissue samples using the Maxwell^®^ 16 Mouse Tail DNA Purification Kit (Promega, Wisconsin, USA), following the manufacturer´s recommendations. The DNA concentration was determined for each sample using a Synergy^TM^ H1 microplate reader (Biotek Instruments Inc, Winooski, VT, USA), and the samples were adjusted to 100 ng/µL in molecular grade water. Parasite DNA detection was carried out by nested PCR adapted to a single tube from the internal transcribed spacer (ITS1) region of *N. caninum* using TgNN1-TgNN2 as external primers and NP1-NP-2 as internal primers [7, 23, 24]. Each reaction was performed in a final volume of 25 µL with 5 µL of sample DNA. PCR was carried out in 9 samples of CA and 9 samples of CO as well as 3 samples of maternal pre-scapular and ileofemoral lymph node and 3 samples of FB and FL. DNA samples from G-Control were included in each round of DNA extraction and PCR as negative controls. Positive PCR controls with *N. caninum* genomic DNA equivalent to 10, 1 and 0.1 tachyzoites in 100 ng of bovine DNA were also included in each batch of amplifications. Fifteen µL aliquots of the PCR products were visualized under UV light in a 1.5% agarose/GelRed™ (Biotium INC) gel to detect the *N. caninum*-specific 247 bp amplification product.

Nested-PCR positive samples were adjusted to a concentration of DNA of 20 ng/µL, and quantification of *N. caninum* DNA was performed by real-time PCR using the equipment ABI 7500 FAST (Applied Biosystems, Foster City, CA, USA). The Nc-5 region was targeted as described elsewhere [25]. A volume of 5 μL of diluted DNA from each sample was used for the qPCR assays. The number of *N. caninum* tachyzoites was determined by interpolating the Ct values (cycle threshold value) on a standard curve. The standard curve was designed for the quantification of 10^-1^ to 10^5^ tachyzoites according to Regidor-Cerrillo et al. [30]. To normalize the quantification of the parasite in each sample, a bovine β-actin standard curve was designed (from 64 ng of DNA per µL to 0.2 ng per µL). The results were expressed as the relation between parasite DNA and cell DNA amount (R^2^ ≥ 0.99; Slope values varied from −3.63 to −3.18).
