# Supplementary figures and images for: Early Neospora caninum infection dynamics in cattle after inoculation at mid-gestation with high (Nc-Spain7)- or low (Nc-Spain1H)-virulence isolates
Source: Vet Res. 2019 Sep 24;50:72. doi: 10.1186/s13567-019-0691-6 (PMC6760050; doi:10.1186/s13567-019-0691-6)

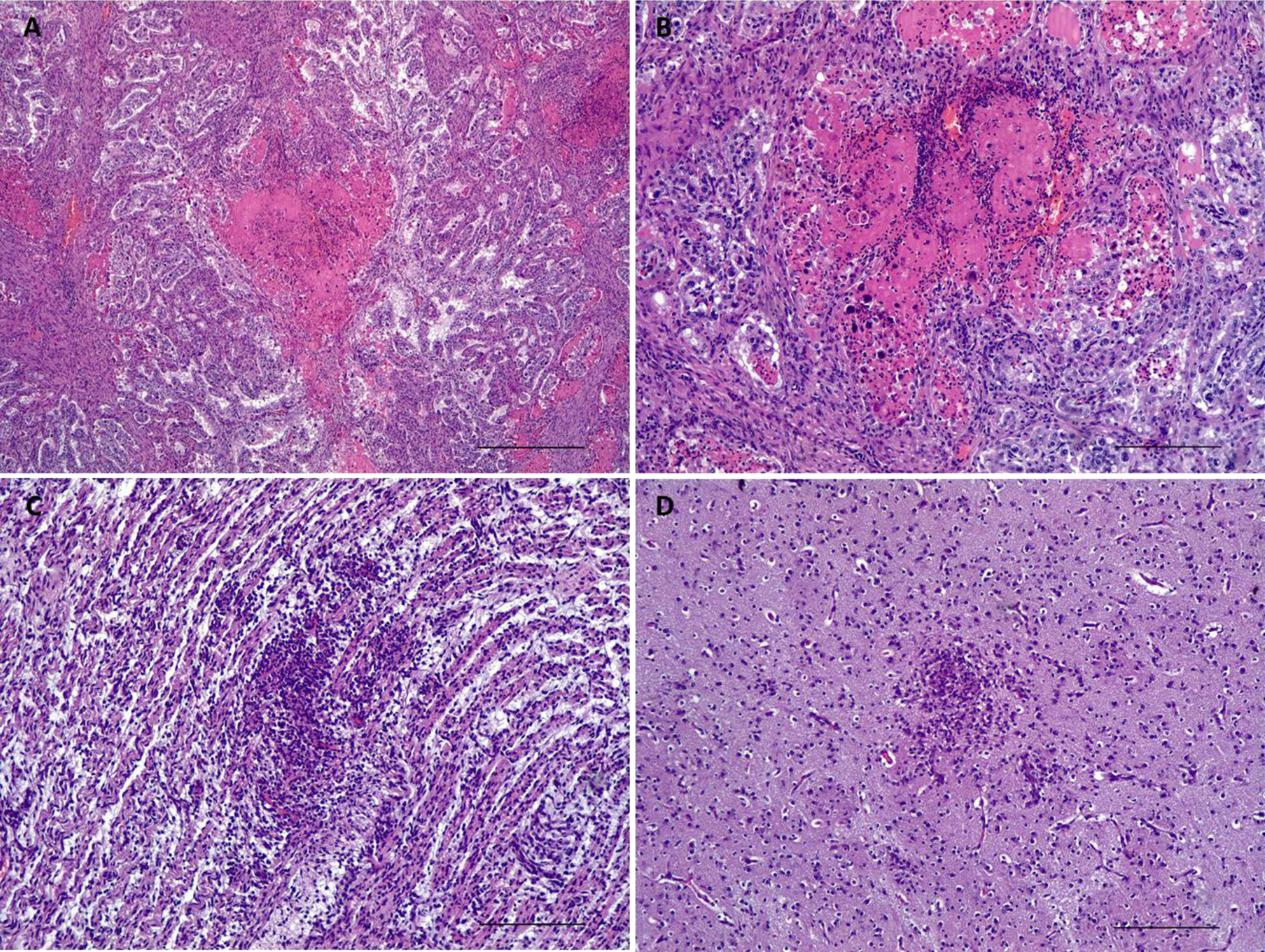

Supplement: Supplementary file 2 — Additional file 2. Histological findings in placental and foetal samples. (A) Placenta. G-NcSpain7 20 dpi. Three foci of necrotic placentitis with mild infiltration of inflammatory cells at the interdigitate area of the placentome HE. 4×. Bar 500 µm. (B) Placenta. G-NcSpain7 20 dpi. Focal necrosis with mild infiltration of inflammatory cells at the interdigitate area of the placentome. HE. 10×. Bar 200 µm. (C) Foetal heart. G-NcSpain7 20 dpi. Focal non-suppurative myocarditis. HE. 10×. Bar 200 µm. (D) Foetal brain. G-NcSpain7 20 dpi. Glia focus with a small area of necrosis at the centre of the lesions. HE. 10×. Bar 200 µm. [file 13567_2019_691_MOESM2_ESM.tif]

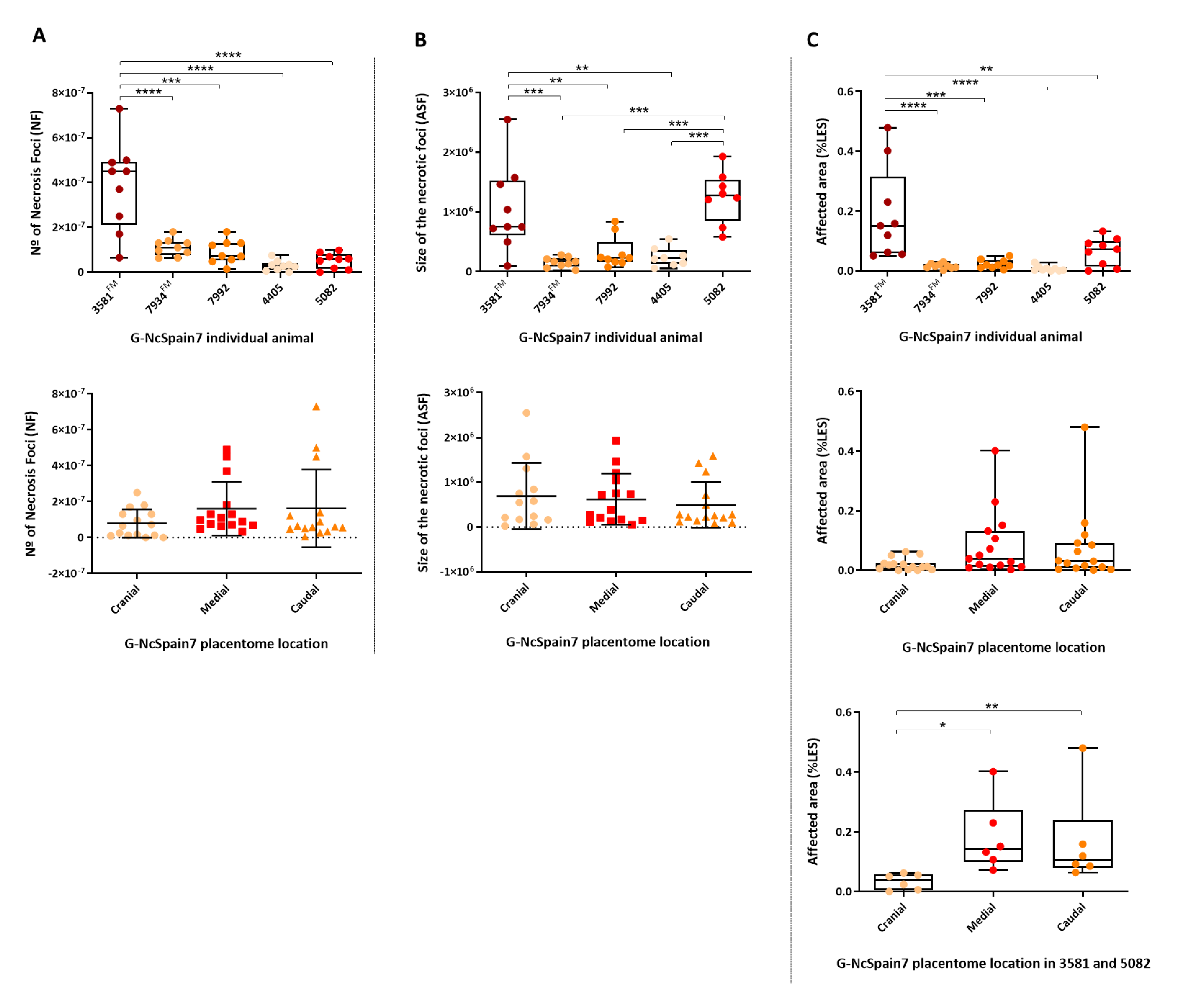

Supplement: Supplementary file 3 — Additional file 3. Quantification of necrosis foci (NF), size (ASF) and affected area (%LES) of these foci. Graphs representing median number of cells, lower and upper quartiles (boxes) and minimum and maximum values (whiskers) of (A) number of NF in G-NcSpain7 culled at 20 dpi studied by individual animal or by placentome location, (B) ASF in G-NcSpain7 culled at 20 dpi studied by individual animal or by placentome location and (C) %LES in G-NcSpain7 culled at 20 dpi studied by individual animal, by placentome location or by placentome location in the placentomes of animals with higher ASF (3581 and 5082). ****, ***, ** and * symbols indicate P < 0.0001, P < 0.001, P < 0.01 and P < 0.05 significant differences. [file 13567_2019_691_MOESM3_ESM.tif]
